# Cross‐inoculation of rhizobiome from a congeneric ruderal plant imparts drought tolerance in maize (*Zea mays*) through changes in root morphology and proteome

**DOI:** 10.1111/tpj.15775

**Published:** 2022-06-08

**Authors:** Ziliang Zhang, Bhupinder Singh Jatana, Barbara J. Campbell, Jasmine Gill, Vidya Suseela, Nishanth Tharayil

**Affiliations:** ^1^ Department of Plant & Environmental Sciences Clemson University Clemson SC USA; ^2^ Department of Biological Sciences Clemson University Clemson SC USA

**Keywords:** *Andropogon virginicus*, cross‐inoculation, drought stress, drought resilience, maize, rhizobiome, root, phytohormones

## Abstract

Rhizobiome confer stress tolerance to ruderal plants, yet their ability to alleviate stress in crops is widely debated, and the associated mechanisms are poorly understood. We monitored the drought tolerance of maize (*Zea mays*) as influenced by the cross‐inoculation of rhizobiota from a congeneric ruderal grass *Andropogon virginicus* (*andropogon‐inoculum*), and rhizobiota from organic farm maintained under mesic condition (*organic‐inoculum*). Across drought treatments (40% field capacity), maize that received *andropogon‐inoculum* produced two‐fold greater biomass. This drought tolerance translated to a similar leaf metabolomic composition as that of the well‐watered control (80% field capacity) and reduced oxidative damage, despite a lower activity of antioxidant enzymes. At a morphological‐level, drought tolerance was associated with an increase in specific root length and surface area facilitated by the homeostasis of phytohormones promoting root branching. At a proteome‐level, the drought tolerance was associated with upregulation of proteins related to glutathione metabolism and endoplasmic reticulum‐associated degradation process. Fungal taxa belonging to Ascomycota, Mortierellomycota, Archaeorhizomycetes, Dothideomycetes, and Agaricomycetes in *andropogon‐inoculum* were identified as potential indicators of drought tolerance. Our study provides a mechanistic understanding of the rhizobiome‐facilitated drought tolerance and demonstrates a better path to utilize plant–rhizobiome associations to enhance drought tolerance in crops.

## Significance Statement

The rhizobiome from a drought‐tolerant ruderal plant (*Andropogon virginicus*) alleviates drought‐induced physiological stress of maize (*Zea mays*) through modifications in root morphology driven by homeostasis of phytohormones and stress‐related proteome upregulations. Thus our study captures the underlying mechanisms through which rhizobiome facilitates drought tolerance in maize, and highlight the importance of cross‐inoculation of rhizobiota from stress‐resilient cogeneric species.

## INTRODUCTION

Climate change and associated abiotic stresses, including drought, flooding, salinity, extreme temperatures, and nutrient deficiency (Hussain et al., 2012; Qin et al., 2016; Rolli et al., 2015), represent a significant threat to agricultural productivity and food security worldwide (Capell et al., 2004; Di Benedetto et al., 2017). Drought is among the most destructive abiotic stresses, which is projected to affect 50% of the arable lands adversely by 2050 (Kasim et al., 2013; Marasco et al., 2013). Owing to the dire consequence of drought‐induced lower crop productivity, extensive research is being carried out worldwide to develop strategies to enhance drought tolerance in crops. Such efforts include screening for drought‐tolerant varieties using conventional breeding or biotechnological approaches, shifting the crop calendars, and improving resource management practices (Venkateswarlu & Shanker, 2009; Vurukonda et al., 2016). Along with the above approaches, the precise modulation of soil microbial communities inhabiting the rhizosphere (rhizobiome) is a promising avenue for improving the performance of crops under environmental stress (Qi et al., 2021).

There is now increasing recognition that plant‐associated microbial communities can significantly stimulate plant growth and enhance plant resistance to abiotic stresses (Mapelli et al., 2013; Qi et al., 2021; Vejan et al., 2016). Sustained by root exudates (Chai & Schachtman, 2022; Sasse et al., 2018; Huang et al. 2019), and root chemistry (Wang et al., 2015), a plethora of microorganisms inhabit rhizosphere forming a complex ecological community that influences plant growth and productivity (Lugtenberg & Kamilova, 2009; Zhalnina et al., 2018). The rhizobiome plays a significant role in plant health, effectively serving as a second plant genome (Berendsen et al., 2012). Beneficial soil rhizosphere microorganisms such as plant growth‐promoting rhizobacteria and/or mycorrhizal fungi can adapt to specific environmental conditions (Kivlin et al., 2013; Marulanda et al., 2009; Qin et al., 2016). Besides adapting themselves to cope with stress, microorganisms can also impart some degree of stress tolerance in plants to sustain the carbon flow to the rhizosphere. Recent studies have highlighted the potential of beneficial microbial strains surviving at extreme environmental conditions to confer drought tolerance to plants (Bainard et al., 2013; Kaushal & Wani, 2016; Sandhya et al., 2010). For example, the bacterium *Achomobacter piechaudii* isolated from dry riverbeds, is capable of increasing salt and drought resistance in both pepper and tomato (Mayak et al., 2004), and the inoculation of *Azotobacter* strains isolated from semi‐arid regions alleviated the drought stress in maize (Shirinbayan et al., 2019).

Despite the well‐known role of rhizosphere microorganisms in conferring drought tolerance to their host plants, current studies have focused primarily on the interactions between plants and individual microbial strains (Kandula et al., 2015; Lebeis et al., 2012). Such approaches have met with variable success, which could be attributed to the complexity in regulating microbial community composition in agricultural soils (Busby et al., 2017), and the potential inability of a non‐rhizobiota to adapt functionally to the rhizosphere of crop plants. Compared with individual microbial species, in natural environments, the plants are more influenced by their interaction with the microbial community. For example, mycorrhizal fungi in natural environments synergize in function with bacterial communities (Bahram et al., 2011), which facilitate water and nutrient foraging. Thus, the biased selection of specific microbial strains over other may compromise any beneficial microbial attributes (e.g., drought tolerance) that are provided by the microbial community. Therefore, it is plausible that microbial‐induced stress tolerance of host plants benefits more from the microbial consortia with greater functional diversity than from a single/few microbial species.

Marginal environments are defined as the areas where salinization of land, availability of nutrients and water, and soil erosion restrict potential crop production (Qureshi, 2017). Ruderal plant species that are uniquely adapted to circumvent or withstand these adverse conditions often thrive in these inhospitable environments (Putnam et al., 1993), and are often characterized by high drought or salt tolerance (Heydarian et al., 2016; Li et al., 2016). Part of this abiotic stress tolerance could be imparted by the beneficial rhizobiome, whose diversity and function are also highly influenced by environmental conditions (Torsvik & Øvreås, 2002). Thus, these ruderal plants thriving in marginal lands could be an ideal candidate for screening for rhizobiome that can confer drought resilience to host plants. Despite the assumed high potential, the effectiveness of consortia of beneficial rhizobiomes from ruderal plants to effectively cross‐infect crop plants and provide stress resilience remains largely unknown, so does any potential mechanism that facilitates stress tolerance.

We hypothesized that cross‐inoculation of the rhizobiome from a stressed environment, rather than from an optimal growing environment, would better equip the crop plants to tolerate the environmental stress. Roots are the initial perceivers of water deficiency signaling, which are critical components of plants to cope with drought stress (Opitz et al., 2016; Yang et al., 2020). Thus, we further hypothesized that the stress tolerance of the crops conferred by rhizobiome inoculation could be achieved primarily through mediating root morphological and metabolic traits. To test these hypotheses, we selected a ruderal plant, *Andropogon virginicus*, to investigate the potential of cross‐inoculation of rhizobiomes in alleviating drought stress of maize (*Zea mays*), one of the major staple crops throughout the world that is highly susceptible to drought stress. *Andropogon virginicus* is a native, herbaceous, perennial, warm‐season bunchgrass colonizing extreme edaphic environments in the eastern United States (Ezaki et al., 2008). It is highly adaptable to poor soil and tolerant to high heat, nutrient‐poor, and drought conditions. In addition, *A. virginicus* is a C4 plant and shares the same subfamily (i.e., Andropogoneae or sorghum tribe) with many important crops such as maize, sugarcane, and sorghum (Brown & Gracen, 1972). This phylogenetic relatedness makes it a potential candidate for relatively easier transfer of its rhizobiome to major cogeneric crops. We examined the influence of cross‐inoculation of rhizobiome from *A. virginicus* (hereafter *andropogon‐inoculum*) and a mesic organic field (hereafter *organic‐inoculum*) on the growth and physiological response of maize under drought stress. We predicted that maize treated with *andropogon‐inoculum* rather than *organic‐inoculum* would show better growth performance and physiology under drought conditions, and this drought tolerance would be driven by changed root system architecture and cellular metabolism.

## RESULTS

### Effect of rhizobiome inoculation on the growth performance of maize under drought stress

**Figure 1:**
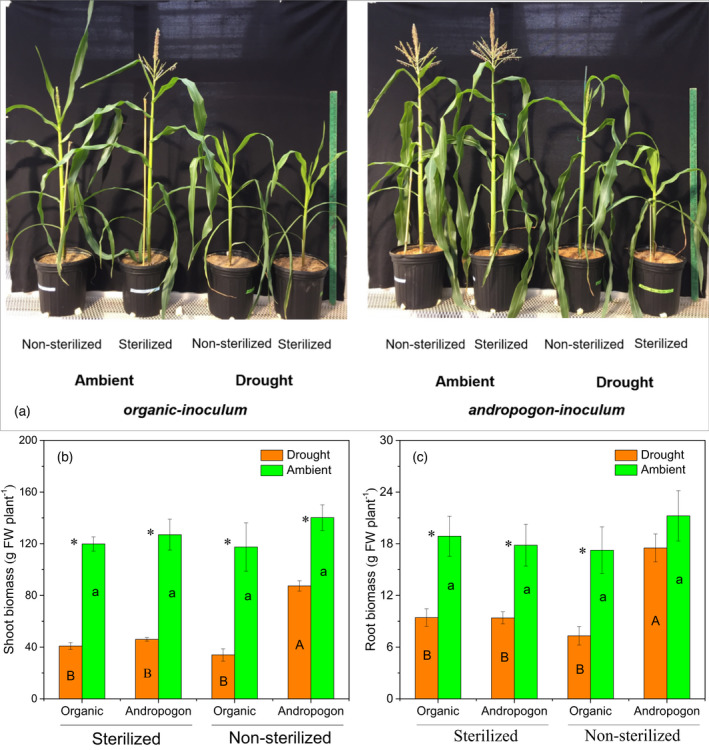
Growth performance of maize under ambient and droughted soil moisture as influenced by cross‐inoculation with various rhizobiome. Growth performance of maize at harvest (35 days after drought treatment) (a), and the shoot (b) and root (c) biomass of maize treated with *andropogon‐inoculum* and *organic‐inoculum* under drought and ambient soil moisture. Error bars are ±1SE of the mean (*n* = 5) with the asterisk indicating significant differences between drought and ambient treatments at *P* < 0.05. Lowercase letters within bars indicate significant differences among all the inoculation treatments under ambient conditions, while uppercase letters indicate significant differences among all the inoculation treatments under drought conditions at *P* < 0.05.

From the initial 60% field capacity (FC), the respective soil moisture reduction to 40% FC (drought) was achieved within 4 days of treatment initiation (Figure S2). Thirty‐five days after drought treatment, drought stress negatively affected the growth of maize seedlings as indicated by reduced shoot height (Figure 1a) and shoot and root biomass (Figure 1b,c) across all inoculation treatments (Table S2). However, maize treated with *andropogon‐inoculum* showed better plant growth under drought conditions as compared with respective sterilized controls and maize treated with *organic‐inoculum*. In particular, drought decreased the shoot biomass of the maize treated with *andropogon‐inoculum* by 37.6% compared with a greater reduction of 71.1% when the maize was inoculated with *organic‐inoculum* (Figure 1b,c).

### Changes of physiological and biochemical properties in maize leaves under drought stress

**Figure 2:**
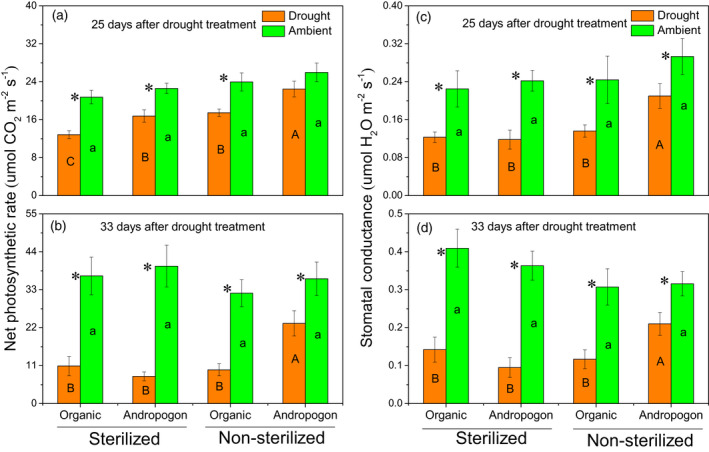
Foliar photosynthetic traits of maize under ambient and droughted soil moisture as influenced by cross‐inoculation with various rhizobiome. (a,b) Net photosynthetic rate and (c,d) stomatal conductance of maize leaves in 25 days and 33 days after drought treatment. Error bars are ±1SE of the mean (*n* = 5) with the asterisk indicating significant differences between drought and ambient treatments at *P* < 0.05. Lowercase letters within bars indicate significant differences among all the inoculation treatments under ambient conditions, while uppercase letters indicate significant differences among all the inoculation treatments under drought conditions at *P* < 0.05.

At 25 days of drought treatment, the foliar net photosynthetic rate (Pn) of the maize treated with *andropogon‐inoculum* was similar to the respective ambient treatment, whereas the Pn of the maize inoculated with *organic‐inoculum* exposed to drought was 27.1% lower than that of the respective ambient treatment (Figure 2a). At 33 days of drought treatment, drought stress significantly decreased Pn of the maize inoculated with both rhizosphere soils; however, the reduction was less for *andropogon‐inoculum* (35.8%) than *organic‐inoculum* (69.4%) (Figure 2b). The responses of stomatal conductance (Gs) were similar to the changes observed in Pn after drought stress, with the decreases in Gs of the maize treated with *andropogon‐inoculum* (28.4% and 33.6% reductions for 25 and 33 days of drought stress, respectively) less than those observed in the maize inoculated with *organic‐inoculum* (44.2% and 62.0% reductions for 25 and 33 days of drought stress, respectively) (Figure 2c and 2d). Compared with sterilized controls, the maize treated with *andropogon‐inoculum* had greater Pn and Gs upon 25 and 33 days of drought (Figure 2). In contrast, no significant difference in the response of Pn and Gs to drought stress was observed between *organic‐inoculum* and respective sterilized controls (Figure 2).

**Figure 3:**
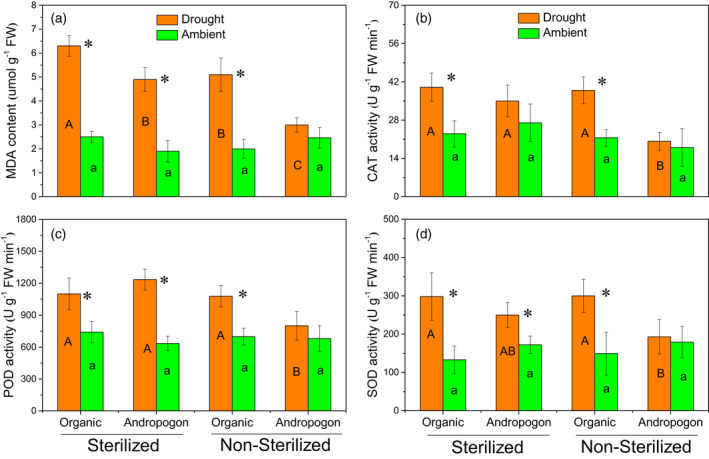
Activities of antioxidant enzymes in leaves of maize under ambient and droughted soil moisture as influenced by cross‐inoculation with various rhizobiome. (a) Malondialdehyde (MDA) content and activities of (b) catalase (CAT), (c) peroxidase (POD), and (d) superoxide dismutase (SOD) in leaves of maize treated with *andropogon‐inoculum* and *organic‐inoculum* under drought and ambient conditions. Error bars are ±1SE of the mean (*n* = 5) with the asterisk indicating significant differences between drought and ambient treatments at *P* < 0.05. Lowercase letters within bars indicate significant differences among all the inoculation treatments under ambient conditions, while uppercase letters indicate significant differences among all the inoculation treatments under drought conditions at *P* < 0.05.

As captured by the malondialdehyde (MDA) level, drought stress did not increase oxidative stress of the maize treated with *andropogon‐inoculum*, but significantly increased oxidative stress of the maize inoculated with *organic‐inoculum* by 153.9% (Figure 3a). Similarly, drought significantly increased activities of catalase (CAT), peroxidase (POD), and superoxide dismutase (SOD) of the maize inoculated with *organic‐inoculum* by 80.6%, 54.4%, and 101.3%, respectively, while it did not influence these antioxidant enzymes of the maize treated with *andropogon‐inoculum* (Figure 3b–d). Compared with sterilized controls, the maize treated with *andropogon‐inoculum* had lower activities of antioxidant enzymes under drought conditions. In contrast, there was no significant difference of enzyme activities between maize that received *organic‐inoculum* and sterilized controls (Figure 3).

### Changes of primary metabolite profiles of maize leaves under drought stress

**Figure 4:**
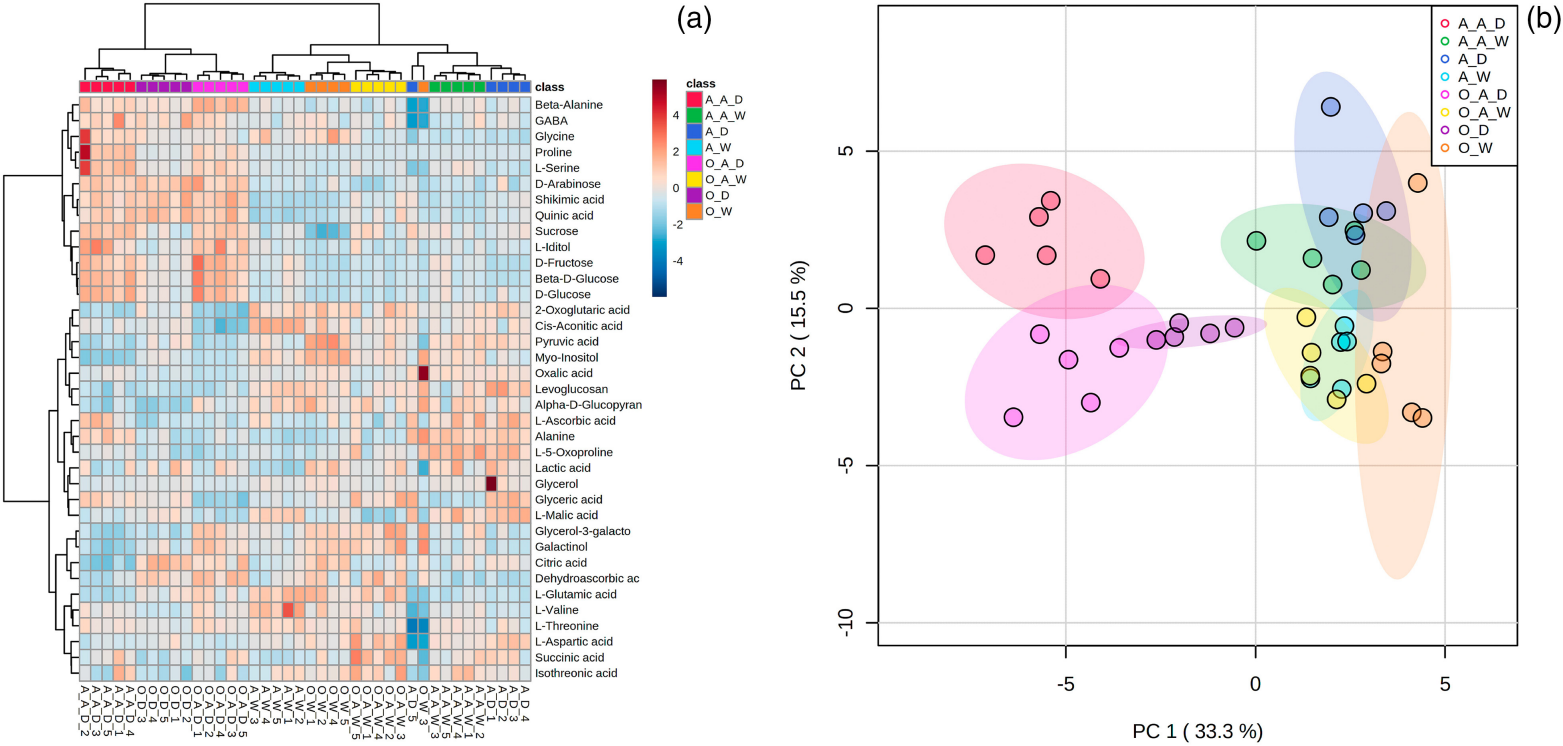
Primary metabolite profiles of maize leaves under ambient and droughted soil moisture as influenced by cross‐inoculation with various rhizobiome. (a) Hierarchical clustering and (b) principal components analyses of differences in primary metabolite profiles of maize leaves among different treatments. **A_A_D**, sterilized *andropogon‐inoculum* under drought condition; **A_A_W**, sterilized *andropogon‐inoculum* under ambient condition; **A_D**, non‐sterilized *andropogon‐inoculum* under drought condition; **A_W**, non‐sterilized *andropogon‐inoculum* under ambient condition; **O_A_D**, sterilized *organic‐inoculum* under drought condition; **O_A_W**, sterilized *organic‐inoculum* under ambient condition; **O_D**, non‐sterilized *organic‐inoculum* under drought condition; **O_W**, non‐sterilized *organic‐inoculum* under ambient condition.

A gas chromatography–mass spectrometry (GC‐MS)‐based metabolomic approach identified 37 compounds in the foliar tissue, including sugars (5), sugar derivatives (9), amino acids (11), and organic acids (12) (Figure 4a). An overview of the variance in the whole data matrix was visualized with an integrated principal components analyses (PCA) plot (Figure 4b). Drought‐induced differences in metabolic products dominated the variances, with drought treatments, except for the non‐sterilized andropogon‐drought treatment, grouped separately from ambient treatments without overlap on PC1, which represented 33.3% of the variation between groups (Figure 4b). Compounds that increased or decreased in response to drought were similar for *organic‐inoculum* and sterilized controls (Figure 4a). The non‐sterilized andropogon‐drought treatment was similar in composition of primary metabolites as that of all ambient treatments (Figure 4b).

### Changes of root architecture traits of maize under drought stress

**Figure 5:**
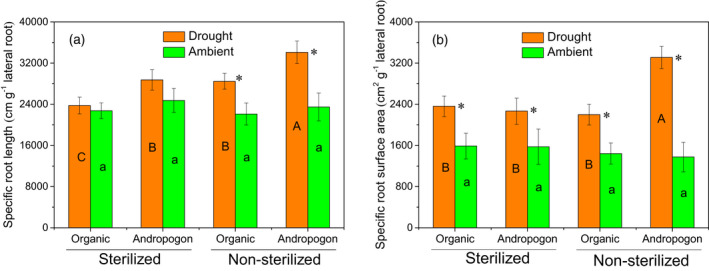
Root architecture traits of maize under ambient and droughted soil moisture as influenced by cross‐inoculation with various rhizobiome. (a) Specific root length and (b) specific root surface area of lateral roots of the maize treated with *andropogon‐inoculum* and *organic‐inoculum* under drought and ambient conditions. Error bars are ±1SE of the mean (*n* = 5) with the asterisk indicating significant differences between drought and ambient treatments at *P* < 0.05. Lowercase letters within bars indicate significant differences among all the inoculation treatments under ambient conditions, while uppercase letters indicate significant differences among all the inoculation treatments under drought conditions at *P* < 0.05.

Drought did not significantly change the specific root length (SRL) and specific root surface area (SRSA) of both primary roots and crown roots of maize in inoculated treatments, while it increased the SRL and SRSA of lateral roots (Figure 5). However, the magnitude of the increase was different between soil inoculations. Specifically, drought significantly increased the SRSA of lateral roots of the maize treated with *andropogon‐inoculum* by 141.0% compared with a lower increment of 52.5% when the maize was inoculated with *organic‐inoculum* (Figure 5b).

### Changes in root hormones of maize under drought stress

Liquid chromatography (LC)‐MS/MS–based analysis identified 23 hormones/precursors across maize root samples of which 19 compounds showed the treatment effect. Along the Axis‐1 of the partial least squares‐discriminant analysis (PLS‐DA) plot that explained 76% of variation, *andropogon‐inoculum* under drought and wet treatments grouped together, and compared with the *sterilized andropogon‐inoculum treatment*, had a lower abundance of tryptamine, topline riboside, tryptophan, and abscisic acid (Figure S4a, b). Compared with the *andropogon‐inoculum* under wet treatment, the *andropogon‐i‐noculum* under drought treatment had a higher abundance of salicylic acid, indole‐3‐acetic acid, zeatin glucoside, and isopentenyladenine glucoside (Figure S4).

### Changes of protein profiles of maize roots under drought stress

**Figure 6:**
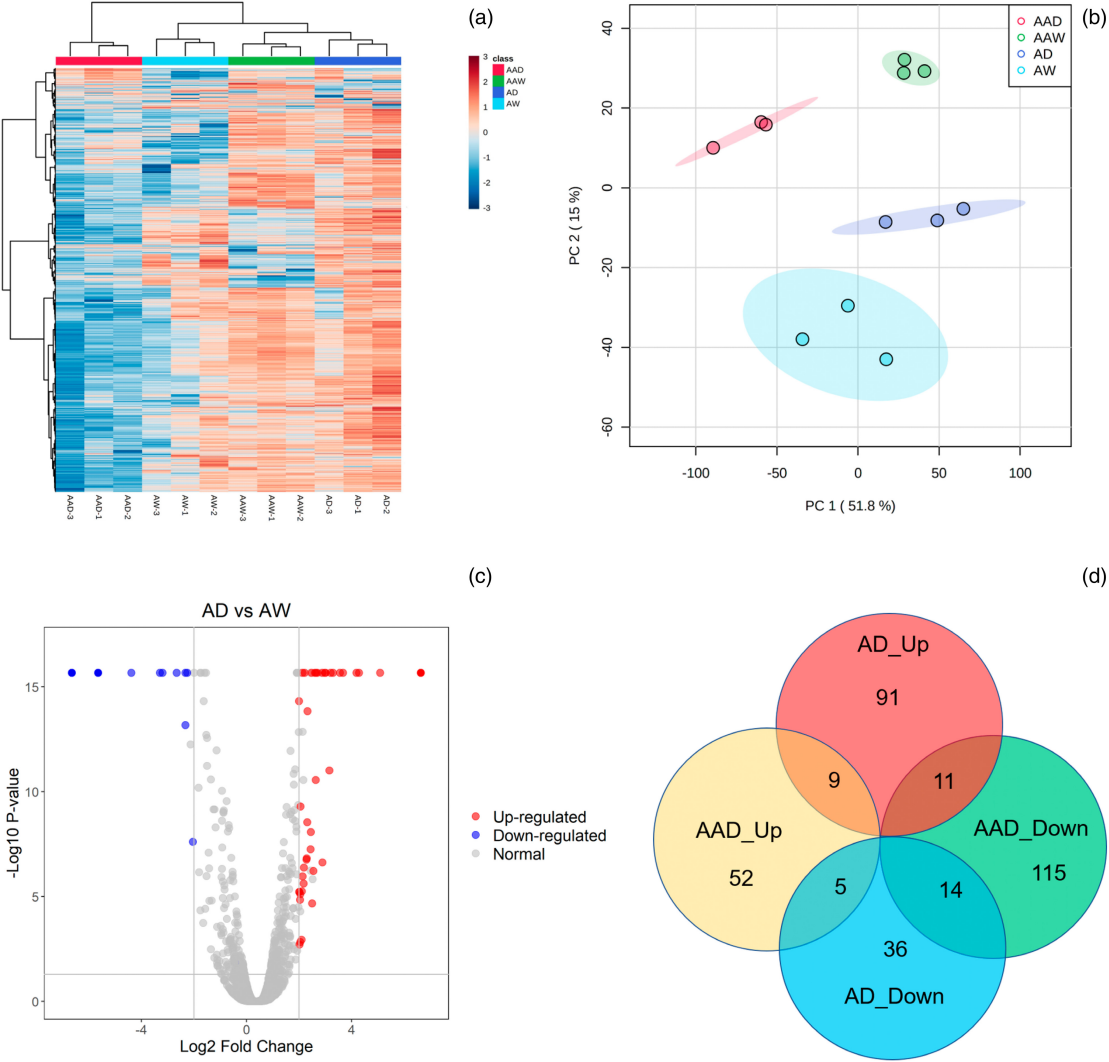
Protein profiles of maize roots under ambient and droughted soil moisture as influenced by cross‐inoculation with various rhizobiome . (a) Hierarchical clustering and (b) principal components analyses of differences in protein profiles of maize roots among different treatments. (c) Volcano plot displaying different expressed proteins between AD and AW treatments. (d) Venn diagram of the distribution of up‐ and downregulated proteins across different treatments. In (c), the red and blue dots represent the upregulated and downregulated proteins between AD and AW treatments, respectively. **AAD**, sterilized *andropogon‐inoculum* under drought condition; **AAW**, sterilized *andropogon‐inoculum* under ambient condition; **AD**, non‐sterilized *andropogon‐inoculum* under drought condition; **AW**, non‐sterilized *andropogon‐inoculum* under ambient condition.

The proteomics approach resulted in reliable identification of 5969 proteins (Figure 6a). An overview of the variance in the whole data matrix was visualized with an integrated PCA plot (Figure 6b). Protein profiles were clearly separated by different treatments for the *andropogon‐inoculum*, which accounted for, in total, 66.8% of explained variance (Figure 6b). Based on the log2 fold‐change >2 (up) or <−2 (down) and a *P* < 0.05 (Figure 6c), 363 significant differentially abundant proteins were identified (Figure 6d). Among these proteins, 102 and 41 differentially abundant proteins were exclusively upregulated and downregulated in non‐sterilized *andropogon‐inoculum* under drought conditions compared with ambient conditions (Figure 6d).

**Figure 7:**
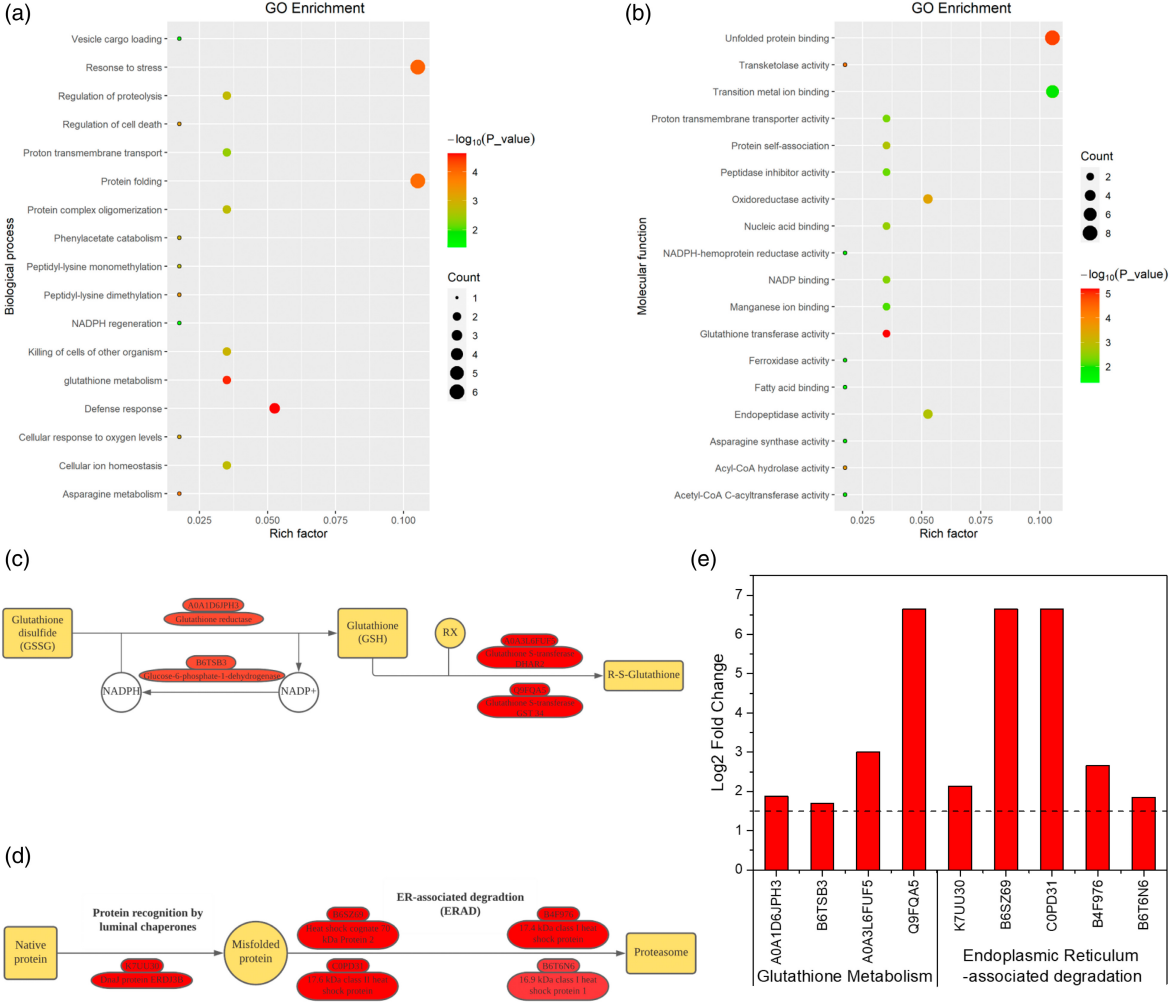
Enriched functional proteins in maize roots under ambient and droughted soil moisture as influenced by cross‐inoculation with various rhizobiome. Gene Ontology (GO) enrichment summarizing the subcategories of differentially abundant proteins detected in maize roots between non‐sterilized *andropogon‐inoculum* under drought condition and non‐sterilized *andropogon‐inoculum* under ambient condition treatments based on (a) biological process and (b) molecular function. The bubble size represents the number of differentially abundant proteins, and the bubble color represents the –log10 (*P* value). (c) Simplified representation of the Kyoto Encyclopedia of Genes and Genomes glutathione metabolism pathway, and (d) protein processing in endoplasmic reticulum pathway. Red boxes represent the upregulated proteins in maize roots treated with *andropogon‐inoculum* under drought stress. Yellow boxes represent related precursors and products. (e) Significance (log2 fold‐change) of related differentially abundant proteins involved in glutathione metabolism pathway and protein processing in the endoplasmic reticulum pathway.

Gene Ontology (GO) enrichment analysis identified 35 functional subcategories of proteins and two primary categories: biological processes and molecular functions (Figure 7a,b). Among the biological process, the expressed proteins in maize roots treated with *andropogon‐inoculum* in response to drought were strongly enriched for GO terms associated with stress response, defense response, protein folding, and glutathione (GSH) metabolism (Figure 7a). In the molecular function category, GO terms associated with unfolded protein binding, oxidoreductase activity, and glutathione transferase activity were enriched (Figure 7b). Pathway enrichment analysis indicated that GSH metabolism and endoplasmic reticulum (ER)‐associated degradation (ERAD) were significantly enriched (Figure 7c,d; *P* < 0.05). Proteins related to GSH metabolism (A0A1D6JPH3, B6TSB3, A0A3L6FUF5, and Q9FQA5) and protein processing in ER (K7UU30, B6SZ69, C0PD31, B4F976, and B6T6N6) were increased in abundance in response to drought stress (Figure 7e).

### Shifts of rhizosphere soil microbial communities under drought stress

The patterns of bacterial and fungal beta‐diversity indicated distinct microbial community compositions between drought and ambient treatments as well as between two soil inoculations (Figure S5). The PC 1 (37% explained variance) showed that the bacterial communities were different between drought and ambient conditions. The PC 2 (33% explained variance) showed that the bacterial communities were different between treatments of *andropogon‐inoculum* and *organic‐inoculum* (Figure S5a). Fungal communities were clearly separated by soil inoculation along PC 1 (43% explained variance), while the difference between drought and ambient conditions for the *andropogon‐inoculum* was primarily represented along PC 2 (7.1% explained variance) (Figure S5b). There was no clear separation of fungal communities between drought and ambient conditions for the *organic‐inoculum* (Figure S5b).

**Figure 8:**
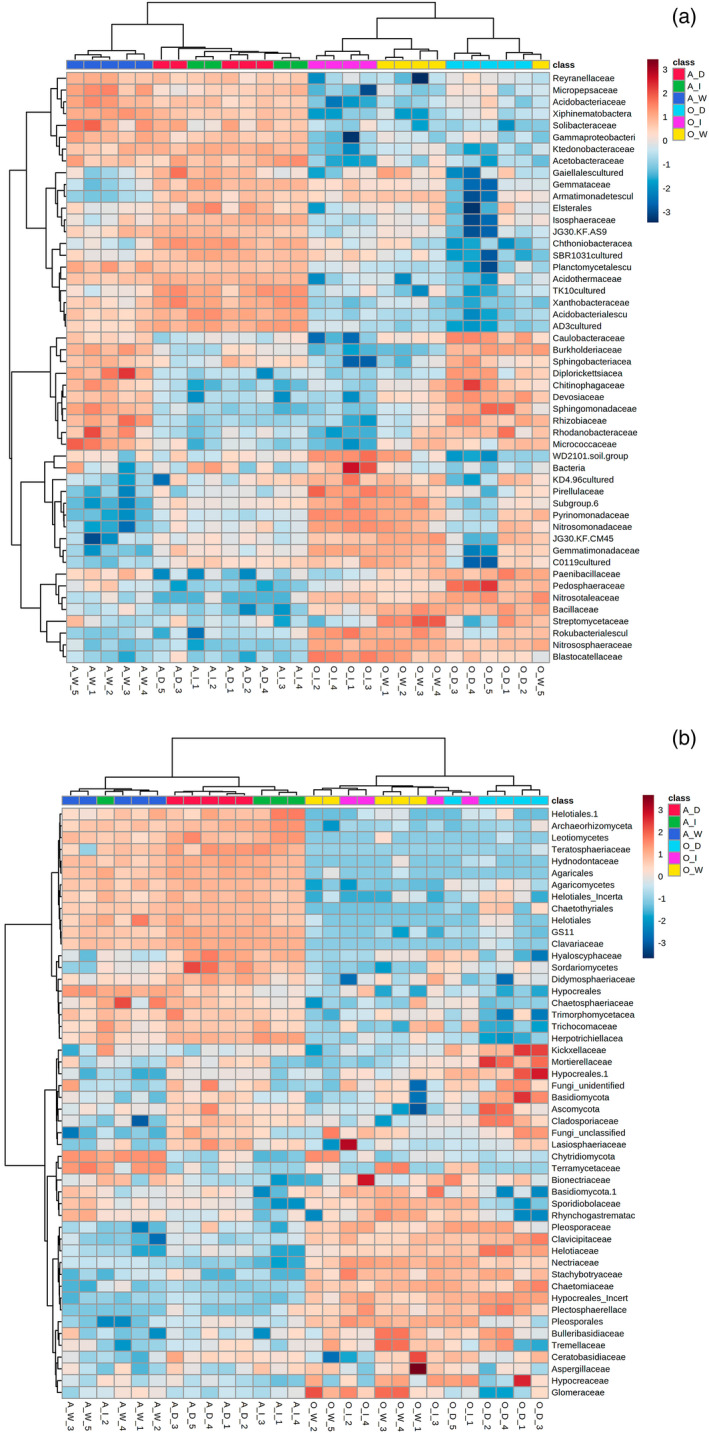
The microbial communities of the initial inoculum and that of maize rhizosphere at the time of harvest. Heatmap showing major (a) bacterial and (b) fungal families with the relative abundance of top 50 in all samples. Colors are scaled from highest (red) to lowest (blue) values within columns based on the logarithmic normalization of the relative abundance of each amplicon sequence variant at the family level. **A_I**, Andropogon soil before inoculation; **A_D**, non‐sterilized *andropogon‐inoculum* under drought condition; **A_W**, non‐sterilized *andropogon‐inoculum* under ambient condition; **O_I**, organic soil before inoculation; **O_D**, non‐sterilized *organic‐inoculum* under drought condition; **O_W**, non‐sterilized *organic‐inoculum* under ambient condition.

Hierarchical cluster analysis of microbial community at the family level revealed that bacterial and fungal communities of *andropogon‐inoculum* significantly differed from those of *organic‐inoculum*, before and after inoculation (Figure 8). Under drought conditions, the *andropogon‐inoculum* had similar bacterial and fungal communities with the initial Andropogon soil (before inoculation). By comparison, the *organic‐inoculum* under ambient conditions had similar microbial communities as the initial organic soil (Figure 8).

**Figure 9:**
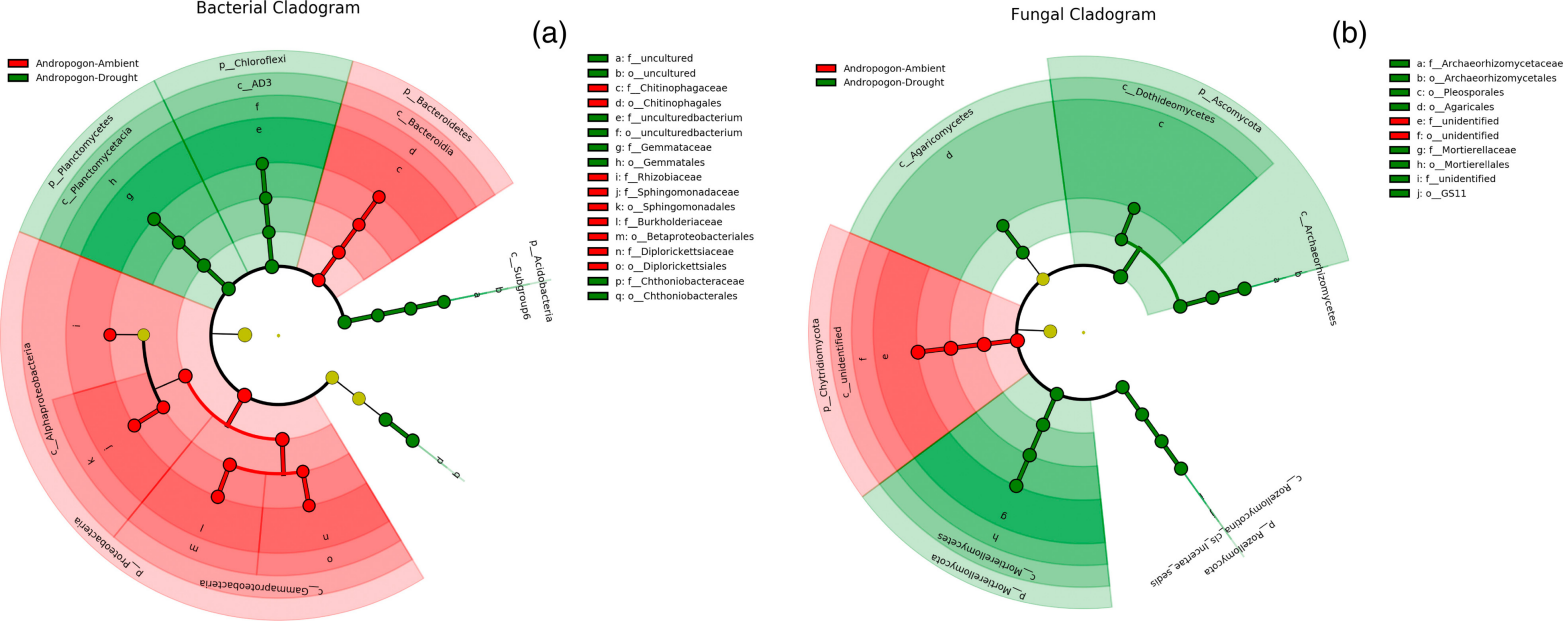
Enriched rhizosphere soil microbial community of maize under ambient and droughted soil moisture as influenced by cross‐inoculation with various rhizobiome. Linear discriminant analysis effect size taxonomic cladogram highlights the (a) bacterial and (b) fungal biomarkers that statistically and biologically differentiate treatments. The cutoff value of ≥4.0 used for the linear discriminant analysis is shown. The five rings of the cladogram stand for domain (innermost), phylum, class, order, and family. Small nodes and shading with different colors (red and green) in the diagram represent abundance of those taxa in the respective group. Yellow circles represent non‐significant differences in abundance between treatments for that particular taxonomic group. Most non‐significant nodes have been filtered to simplify the cladogram.

To find specific bacterial and fungal groups enriched within the treatments of *andropogon‐inoculum* under drought conditions, the linear discriminant analysis effect size (LEfSe) method was performed from phylum to family level according to two different grouping patterns (Figure 9). For bacteria, compared with the ambient conditions, the relative abundances of three phyla (Chloroflexi, Planctomycetes, and Acidobacteria), one class (Planctomycetacia), two orders (*Gemmatales* and *Chthoniobacterales*), and two families (*Gemmataceae* and *Chthoniobacteraceae*) were enriched in *andropogon‐inoculum* under drought conditions (Figure 9a). For fungi, the relative abundances of three phyla (Ascomycota, Rozellomycota, and Mortierellomycota), five classes (Archaeorhizomycetes, Rozellomycotina, Mortierellomycetes, Agaricomycetes, and Dothideomycetes), four orders (*Archaeorhizomycetales*, *Mortierellales*, *Agaricales*, and *Pleosporales*), and two families (*Archaeorhizomycetaceae* and *Mortierellaceae*) were significantly higher in *andropogon‐inoculum* under drought than ambient conditions (Figure 9b).

## DISCUSSION

The association of plants with rhizosphere soil microbial communities is now recognized as a conduit that enables plants to inhabit environments that are otherwise inhospitable (Egamberdiyeva & Höflich, 2003; Timmusk et al., 2014). Utilization of such associations in agriculture is a highly promising and sustainable avenue to enhance crop production in marginal lands. In this work, we showed that the rhizobiome from *A. virginicus* adapted to stressed environments were able to enhance plant growth and alleviate physiological stress of maize grown under drought conditions, highlighting the importance of soil microbiome from a ruderal plant species in conferring stress tolerance to a phylogenetically related crop species.

### Effect of rhizobiome inoculation from *Andropogon virginicus* on the drought response of maize

Plant responses, as captured by gas exchange, biomass, oxidative damage, foliar metabolite profiles, and enzyme activities, indicated that plants experienced physiological stress in response to water limitation. Consistent with previous studies (Anjum et al., 2017; Ge et al., 2012; Timm et al., 2018), we observed decreases in plant productivity and inhibitions of photosynthetic processes in maize under drought treatment. However, consistent with our first prediction, the severity of drought stress in maize was modulated by rhizobiome inoculation treatments, with the *andropogon‐inoculum*, rather than *organic‐inoculum*, largely mitigating the drought stress. This mitigatory effect of drought stress was evidenced by a lower inhibition on plant growth and photosynthetic activities of maize. Given that this effect was only observed in the non‐sterilized *andropogon‐inoculum* treatment but not in the sterilized control, the observed drought tolerance would be driven by the biotic rather than abiotic components of *andropogon‐inoculum*. It has been reported that soil microbes previously subjected to drought stress could facilitate drought tolerance in plants (Azarbad et al., 2018; Lau & Lennon, 2012). When stress conditions are maintained or repeated, soil microbial communities and functions can become fixed, and will retain their response to a stressor of similar nature (Azarbad et al., 2020; Hawkes & Keitt, 2015). In line with this, it is possible that the *andropogon‐inoculum* that has been collected from marginal growing conditions likely transferred the soil microbial community associated and the functions that evolved under drought to the new host under drought conditions. This reasoning is supported by the high similarity of microbial community composition between the initial *andropogon‐inoculum* collected from the field, and that of the maize rhizosphere that received *andropogon‐inoculum* and was exposed to drought.

The drought mitigation effect imparted by *andropogon‐inoculum* is further reflected in the lower level of oxidative damage (indicated by less MDA accumulation) of maize leaves under drought conditions. Osmotic adjustment and augmented production of antioxidant enzymes have been proposed as two main strategies in plants to withstand the oxidative damage induced by water stress (Abid et al., 2018; Apel & Hirt, 2004; Xu et al., 2016). Beneficial rhizosphere microbes could increase ability of the plant to produce antioxidant enzymes or osmolytes to withstand drought stress (Carlson et al., 2020; Curá et al., 2017; Khan et al., 2019). In this study, however, the drought did not change the level of antioxidant enzymes and osmolytes (indicated by proline and soluble sugars; Figure S3) in leaves of the maize treated with *andropogon‐inoculum*. This indicates that the *andropogon‐inoculum* potentially enables the maize to reduce cellular perturbation to drought, and the mitigation strategy is proactive than reactive in nature. This is further supported by the similarity in primary metabolite profiles in maize leaves between the *andropogon‐inoculum* subjected to drought treatment and all well‐watered treatments, and the lack of proline accumulation in leaves of maize exposed to the former.

### Andropogon‐inoculum induced changes of root architectural, and proteomics could explain the enhanced drought tolerance of maize

Compared with leaves, the root system of plants is more responsive to moisture stress and first perceive the decrease in soil water potential, thus may trigger more effective stress responsive mechanisms to maintain water uptake when associated with beneficial soil microbes (Manzi et al., 2017). Root morphology is highly associated with drought resistance, with longer and more extensive root systems having higher potential to take up water and nutrients (Naylor & Coleman‐Derr, 2018). The accumulated evidence on the beneficial plant–microbe interactions points to microbe‐mediated changes in root growth and architecture (Contesto et al., 2010; Sukumar et al., 2013), particularly the changes in the root length and surface area (German et al., 2000; Molla et al., 2001). Our results showed that the *andropogon‐inoculum* greatly increased the SRL and SRSA of maize roots under drought conditions compared with those under well‐watered conditions, which implied a shift of root architecture towards higher water absorption efficiency. The improved water uptake efficiency derived from microbially mediated changes in root system architecture could sustain the cellular water potential of plants, thus might lower the response of growth and physiological traits of aboveground plant tissues to drought stress. Further, our data indicate that this change in root architecture could be brought about by the changes in phytohormone levels and interactions. The maize roots from the *andropogon‐inoculum* under drought treatment had a higher abundance of salicylic acid, indole‐3‐acetic acid, zeatin glucoside, and isopentenyladenine glucoside, all of which have been shown to alter the root architecture through interactive effects. For example, salicylic acid promotes lateral branching of roots even at femtomolar concentrations (Echevarria‐Machado et al., 2007), primarily by changing the synthesis, transport, and accumulation of auxins (Pasternak et al., 2019), all of which initiates lateral root initiation. The higher accumulation of auxin in the *andropogon‐inoculum* under drought treatments is further evidenced by the upregulation of proteins involved in the auxin biosynthesis and transport. Maintenance of zeatin and isopentenyladenine, the two cytokinin repressors of root growth, in the inactive glycosylated form (Shang et al., 2016), further would increase the sensitivity of root branching to indole‐3‐acetic acid (Meier et al., 2020). The higher abscisic acid content in *sterilized‐drought* treatments reflects physiological stress response that is common under drought stress (Karanja et al., 2021), which could relate to the lower stomatal conductivity in these treatments.

Despite the above morphological and physiological changes that would facilitate an efficient uptake of water that is scarce under drought conditions, physiological water stress is inevitable. However, as documented by the lipid peroxidation, the plants under drought conditions with *andropgon‐inoculum* did not suffer from extensive physiological damage, part of which could be facilitated by the physiological stress mitigation strategies. Apart from the modification of root architecture, beneficial rhizosphere microbes can directly facilitate host plants to activate stress response pathways related to enhance plant drought tolerance (Cho et al., 2013). Our root proteomics analysis revealed that *andropogon‐inoculum* significantly upregulated proteins associated with GSH metabolism and ERAD in maize roots. The GSH metabolism pathway responds to abiotic stress mainly by affecting GSH synthesis (Hasanuzzaman et al., 2017). GSH, acting as a non‐enzymatic antioxidant, has been widely reported to protect plants from the oxidative damages caused by abiotic stresses including drought (Alam et al., 2014; Hasanuzzaman et al., 2017). In our study, the abundance of glutathione *S*‐transferase and glutathione reductase, which are involved in GSH‐mediated reactive oxygen species scavenging, were increased in roots of maize treated with *andropogon‐inoculum* under drought, suggesting that increased expression of glutathione reductase and glutathione *S*‐transferase by *andropogon‐inoculum* may contribute to enhancing plant drought resistance by maintaining GSH pools. Similar to GSH, the ER plays a crucial role in the maintenance of cellular homeostasis through folding and modification of secretory and transmembrane proteins (Liu et al., 2011). Abiotic stresses such as drought can evoke ER stress by accumulating misfolded proteins in the ER (Chen et al., 2019). ERAD is considered to play important roles in restoring ER homeostasis by directly recognizing misfolded proteins and mediating their degradation (Strasser, 2018). Many proteins upregulated by *andropogon‐inoculum* treatment under drought conditions in this study are involved in the ERAD process. These include several heat‐shock proteins (HSPs), molecular chaperones that regulate the folding and degradation of protein molecules, which are believed to impart substantial tolerance to multiple abiotic stresses (Feder & Hofmann, 1999; Pegoraro et al., 2011). For instance, increased accumulation of HSP70 has been reported in the drought‐tolerant barley genotype rather than the drought‐sensitive genotype (Kausar et al., 2013). Transgenic tobacco seedlings that overexpressed HSP70 exhibits tolerance to drought stress (Cho & Choi, 2009). Overall, our data indicated that the drought mitigation effect imparted by *andropogon‐inoculum* might be partly attributed to the upregulated root proteins related to the maintenance of cellular homeostasis, more specifically, GSH metabolism and ERAD process.

### Potential key microbial consortia within andropogon‐inoculum contributing to drought mitigation of maize

Although the inoculation of rhizobiomes from *A. virginicus* greatly reduced the drought stress of maize, a critical question is which microbial communities or groups play dominant roles in facilitating drought mitigation of host plants. By simultaneously surveying the microbial communities assembled under drought and well‐watered conditions at both the beginning and the end of the experiment, we were able to identify the potential key microbial consortia in *andropogon‐inoculum* that were enriched by drought stress. Enriched microbial taxa under drought stress could contribute to higher frequencies of these microbial functional genes within the microbial community (Santos‐Medellín et al., 2017; Treseder et al., 2018), which may also increase the possibility to confer drought tolerance to their host plants. Based on the LEfSe analysis, Ascomycota, Mortierellomycota, Archaeorhizomycetes, Dothideomycetes, and Agaricomycetes were considered the indicator groups in *andropogon‐inoculum* under drought condition. It has been previously reported that various drought‐tolerant, melanized Ascomycota predominated in arid lands (Porras‐Alfaro et al., 2011). In addition, Dothideomycetes have been shown to be associated with increased plant yield under drought (Andreo‐Jimenez et al., 2019). Inoculation of some members of mycorrhiza‐like endophytic Agaricomycetes also provided drought tolerance to host plants exposed to drought stress (Gill et al., 2016; Pérez‐Alonso et al., 2020). We were not able to ascertain the detailed taxonomic response of fungal amplicon sequence variants to drought in the present study due to the large fraction of unclassified taxa. We also found drought‐related shifts in bacterial community compositions in both inocula. In particular, Acidobacteria, Chloroflexi, and Planctomycetes were significantly enriched under drought, whereas Proteobacteria and Bacterodetes were generally depleted. This could be partially explained by the different bacterial cell wall characteristics that have been associated with desiccation tolerance (Santos‐Medellín et al., 2017). In particular, monoderm bacteria such as Chloroflexi with thick cell walls have been shown to be more resistant to water stress (Lennon et al., 2012). However, given that the *organic‐inoculum* did not induce a drought tolerance in maize, these broad taxonomic trends observed across both *andropogon‐inoculum* and *organic‐inoculum* despite their intrinsic compositional differences may suggest a reproducible response of rhizosphere bacterial communities of maize to drought stress. It should be noted that while particular phyla or families have been proposed for containing plant growth promoting strains under drought stress, the traits that benefit plant growth are not necessarily shared among all members of these taxa. Thus, the sequence‐based summary of fungal community composition reported here should be followed up with additional work to screen further for consortia that harbor corresponding functions, which will help to tailor an optimal microbial community conferring maximum benefits to the hosts.

## CONCLUSION

In conclusion, our study showed the high potential of transferring the rhizobiome and the associated function from a ruderal plant to a congeneric crop. The higher drought mitigation effect was associated with the increase in SRL and surface area regulated by the phytohormones and upregulation of proteins related to GSH metabolism and ERAD process in maize roots. Fungal taxa belonging to Ascomycota, Mortierellomycota, Archaeorhizomycetes, Dothideomycetes, and Agaricomycetes in *andropogon‐inoculum* that were upregulated under drought stress could be proposed as potential indicator taxa contributing to drought tolerance of maize. While pairwise plant–microbe combinations are crucial and required for mechanistic studies, the present work represents an important step in understanding the depth and degree to which soil microbial communities interact with plants to impart stress tolerance.

## EXPERIMENTAL PROCEDURES

### Soil collection and characterization

Field soils from two sites were used as rhizobiome inocula in our study, one collected from a marginal land (stressed environment) where *A. virginicus* is growing naturally in Anderson County, South Carolina (34°33′N, 82°40′W, and 232 m a.s.l.), and another sampled from a mesic and fertile site (optimal environment) where maize is cultivated at the Clemson University Student Organic Farm in Clemson, South Carolina (34°40′N, 82°50′W, and 190 m a.s.l.). Both soils are similar in texture and classified as sandy loam. The rhizosphere soil collection was performed according to the modified protocol from McPherson et al. (2018). Briefly, twenty plants were randomly chosen from each sampling site, with each plant being excavated to a depth of 30 cm. The bulk soil was removed from the roots, and the plant was placed in a 5‐gallon bucket and further shaken vigorously to collect the rhizosphere soil that was closely attached to the root surface. The rhizosphere soil was sieved through a 2‐mm sieve to remove any rock and debris. The rhizosphere soil from each plant was combined and thoroughly homogenized into a composite soil. Four subsamples (20 g each) of the composite sample were immediately placed into sterilized 50‐ml centrifuge tubes and frozen in dry ice for initial microbial community analysis. The remaining samples were placed on ice and transported to the lab. Available nitrogen (N), phosphorus (P), and potassium (K) were determined in both soils to characterize the soil nutrient pools (Table S1), which were used to normalize the soil nutrient level in the following greenhouse experiment.

### Greenhouse experiment

#### Inoculum preparation

Growth substrate for this experiment was river sand sterilized by autoclaving three times at 1‐week intervals at 25% volumetric water content. A subset (4 L) of andropogon and organic rhizosphere soil was also autoclaved as above to be used as a control for soil inoculation treatment. Sterilized sand was filled into 52 surface‐sterilized 5.6‐L plastic pots. Inoculation treatment was applied by mixing 1400 g (20% of substrate weight) of either non‐autoclaved or autoclaved soil inoculum. Specifically, four different inoculum treatments were prepared as follows: (i) 80% sterilized sand + 20% sterilized andropogon rhizosphere soil (sterilized *andropogon‐inoculum*); (ii) 80% sterilized sand + 20% non‐sterilized andropogon rhizosphere soil (non‐sterilized *andropogon‐inoculum*); (iii) 80% sterilized sand + 20% sterilized organic rhizosphere soil (sterilized *organic‐inoculum*); and (iv) 80% sterilized sand + 20% non‐sterilized organic rhizosphere soil (non‐sterilized *organic‐inoculum*) (Figure S1). After setting up the pots, the FC of each treatment was computed as per Wehner et al. (2016). The initial FC of the filled pots with *andropogon‐inoculum* and *organic‐inoculum* treatments was similar (20.3% and 19.8%, respectively) on volumetric soil moisture content.

#### Plant material and drought treatment

Maize (*Zea mays*; Allure®) seeds were surface sterilized with 70% ethanol for 3 min, soaked with sterilized distilled water overnight, and sowed into 40 pots with different soil inoculum treatments. Before sowing, contents of N, P, and K in each pot were normalized by adding specific amounts of sterilized KNO_3_ and KH_2_PO_4_ solutions per the soil nutrient analysis. Three seeds were sown in each pot, and 1 week after emergence seedlings were thinned to maintain one plant per pot. The real‐time soil moisture was continuously (30 sec) monitored using time‐domain reflectometry (TDR) probes (TDR‐100; Campbell Scientific, Logan, UT, USA) (Figure S2). During the initial growth phase, the soil moisture was adjusted daily to 60 ± 5% FC for 20 days during seedling establishment, based on the water loss recorded by weighing the pots at daily intervals. The drought stress was induced thereafter by increasing the irrigation level to 80 ± 5% FC for the ambient treatment, while reducing the irrigation frequency to achieve 40 ± 5% FC for the drought treatment (Figure S2). The experiment was laid out in a factorial completely randomized design with two irrigation levels including 80 ± 5% FC (ambient) and 40 ± 5% FC (drought) used to induce moisture stress. All the inoculation treatments were replicated five times. A nutrient solution (Hoaglands solution) was added at weekly intervals. The plants were grown in a greenhouse at approximately 26°C with 60% relative humidity under natural sunlight, and the photoperiod was maintained at 16/8 h (day/night) with supplemental lighting at an intensity of 300 μmol m^−2^ sec^−1^.

### Plant and soil sampling

Plants were harvested 55 days after the start of the experiment, and the fresh weight and height of the shoot were recorded immediately. Six fully expanded leaves from each plant were sampled and placed on dry ice and stored in −80°C until used for biochemical analysis. Roots were shaken to remove all bulk soil, and the soil tightly adhering to roots (rhizosphere soil) was collected into a sterilized 50‐ml tube and stored in −80°C for microbial community analysis. After gently cleaning in running water, the soil‐free roots were weighed and then separated into three subsamples. One subsample was kept in distilled water for root morphology measurement that was completed within 24 h, the second subsample was kept in 70% ethanol for root mycorrhizal colonization measurement within a week, and the remaining roots were oven‐dried to a constant weight at 65°C.

### Drought response of shoots

#### Measurement of foliar gas exchange

At 25 and 33 days after the initiation of drought treatment, the foliar net Pn and Gs were determined using the Li‐6400 portable photosynthesis measurement system (model LI‐6400; LI‐COR, Lincoln, NE, USA). These parameters were measured under the following conditions: 800 μmol m^−2^ sec^−1^ photosynthetic photon flux density, 500 μmol sec^−1^ flow rate, leaf temperature 30 ± 2°C, and relative humidity 60 ± 1%. Before measurement, the leaves were illuminated for more than 1 h to maintain stomatal opening. The measurements were taken on the second fully open leaf beneath the top leaf for each plant, and three measurements per leaf were recorded.

#### Measurement of foliar lipid peroxidation and activities of antioxidant enzymes

At the time of harvest, the content of MDA, a marker of oxidative damage of lipids, was determined as described in Liu et al. (2014). Fresh leaves were homogenized in 5% (w/v) trichloroacetic acid and reacted with an equal volume of 0.67% (w/v) thiobarbituric acid in a boiling water bath for 30 min. After cooling to room temperature, the mixture was centrifuged, and the supernatant was measured at 532 nm and corrected for non‐specific turbidity by subtracting the absorbance at 600 and 450 nm.

Activities of enzymatic antioxidants, including SOD, POD, and CAT, were determined according to Aebi (1984),Liu et al. (2014), and Huo et al. (2016). Briefly, 0.5 g of frozen leaves were ground into fine powder in dry ice and homogenized in 5 ml of 50 mm potassium phosphate buffer (pH 7.0) containing 1 mm EDTA and 1% polyvinylpyrrolidone. After centrifugation, the supernatant was used to measure enzyme activities. Total SOD activity was assayed by monitoring the inhibition of photochemical reduction of nitro blue tetrazolium at 560 nm (Liu et al., 2014). CAT activity was measured spectrophotometrically at room temperature by monitoring the decrease in absorbance at 240 nm resulting from the consumption of H_2_O_2_ (Aebi, 1984). POD activity was determined by measuring the increase in absorbance at 420 nm for 4‐methylcatechol (Huo et al., 2016).

#### Primary metabolites profiling in maize leaves

Primary metabolites, mainly as sugars, amino acids, and organic acids present in leaves, were quantified using GC‐MS as per Maroli et al. (2016) and Edayilam et al (2018). Briefly, 200 mg of finely powdered leaf tissues were extracted with 2 ml of methanol in a homogenizer at 4°C (Precellys Evolution with Cryolys® Evolution). The supernatant was transferred into glass tubes, and the polar metabolites were separated by adding an equal volume of ice‐cold chloroform, followed by cold water. Subsamples (10 μl) of the top aqueous‐methanol phase were transferred into glass inserts, and 10 μl of 20 μg ml^−1^ ribitol (internal standard) and 50 μg ml^−1^ of d27‐myristic acid in hexane was added and then completely dried in a vacuum evaporator. Dried samples were methoxylaminated at 40°C with 20 μl of methoxylamine hydrochloride (40 mg ml^−1^) in pyridine for 90 min and further silylated with 90 μl of *N*‐methyl‐*N*‐(trimethylsilyl)trifluoroacetamide with 1% trimethylchlorosilane for 40 min at 40°C. The silylated metabolites were analyzed using an Agilent 7980A GC system coupled to a 5975 C Series Mass Detector (Agilent Technologies, Santa Clara, CA, USA). Metabolite peaks were identified by comparing the mass spectra and retention time index of the sample with that of the Kovats RI library.

### Drought response of roots

#### Measurement of root architecture traits

Morphology and architecture of roots were captured by scanning at 300 dpi using a Hewlett Packard Scan Jet 3c/T optical scanner (Epson Perfection V800 photo). The image was then analyzed with winrhizo software (Regent Instruments, Inc., Quebec, Canada) for the average diameter, total length, and total surface area. Before scanning, roots were physically separated into three different groups (i.e., lateral root, crown root, and primary root) based on the structure of the maize root system. The corresponding root samples were oven‐dried at 65°C for 48 h and weighed. SRL and SRSA were calculated as the root length per unit dry weight and root surface area per unit dry weight, respectively (Comas & Eissenstat, 2009).

#### Root hormone analysis

Hormones in roots of the maize subjected to *andropogon‐inoculum* were profiled using a targeted metabolomics approach as described in Simura et al. (2018). Briefly, 20 mg of freeze‐dried roots were extracted twice with 1 ml of 50% acetonitrile using the bead‐beating homogenization described above. To the pooled extract 5 ng of trans‐zeatin (d5) was added as the internal standard. The extract was cleaned up using hydrophilic–lipophilic‐balanced solid phase extraction cartridge. The flow‐through from the cartridge that contains the phytohormones were collected, dried down under nitrogen, and reconstituted in 80 μl of 50% methanol. The samples were analyzed for 101 phytohormones using a liquid chromatograph coupled to a triple quadrupole mass spectrometer (LCMS 8040; Shimadzu Scientific, Columbia, MD, USA) using the ion transitions reported in Simura et al. (2018).

#### Proteomics analysis of maize roots

Briefly, 50 mg freeze‐dried root samples were ground with liquid nitrogen into powder, and then homogenized in 1 ml of extraction buffer containing 100 mm triethylamonium bicarbonate (TEAB) pH adjusted to 8.5 with 12% H_3_PO_4_, 3% sodium dodecyl sulfate, 20 mm Tris(2‐carboxyethyl) phosphine hydrochloride, and 1× EDTA‐free Halt Protease inhibitor. The protein extraction was facilitated by sonicating the samples in ice for 2 min. Protein reduction was performed by incubating the extract at 95°C for 15 min. After cooling to room temperature the extracts were further alkylated by adding indole acetamide (40 mm), and incubating in darkness for 30 min. Protein content was determined using the Qubit Protein assay kit (Garcia et al., 2017) on a Qubit™ 4 Fluorometer (Invitrogen, Thermo Fisher Scientific Inc., USA). Removal of sodium dodecyl sulfate from the extract and the digestion of the alkylated proteins were done on the Suspension Trap (Protifi, Huntington, NY, USA) following the manufacturer's procedures. Briefly, extract equivalent to 150 μg of proteins was mixed with binding buffer (90% methanol; 100 mm TEAB; pH 7.1) and loaded at 500 μl aliquots to a Suspension Trap filter, spun at 2000 rpm for 0.5–1 min, and the flow‐through was discarded. The protein aggregates that are retained on the filter were washed five times with the binding buffer. Finally, 10 μg of sequencing‐grade trypsin dissolved in 200 μl of 50 mm TEAB were added into the filter, centrifuged briefly, the flow‐through returned to the trap, and digested at 37°C for 12 h. Peptides were eluted by stepwise addition and centrifugation of 80 μl of three buffers: 50 mm TEAB, 0.2% formic acid in H_2_O, and 50% acetonitrile and 0.2% formic acid in H_2_O. The peptide eluates were pooled, dried under vacuum, and reconstituted in 50 μl of 0.1% formic acid, and transferred to low‐volume vials for analysis.

The protein extracts were analyzed on an Ultimate 3000 HPLC (Thermo Scientific, Waltham, MA, USA) coupled with an Orbitrap Fusion Tribrid mass spectrometer (Thermo Scientific) using direct injection. Sample equivalent of 1 μg of protein was loaded on to a PepMap™ RSLC C18 nano‐LC columns (2 μm, 100A, 75 μm × 50 cm; Thermo Scientific) coupled to a nano‐ESI (EASY‐Spray™; Thermo Scientific). The peptides were separated using a gradient elution of 0.1% formic acid and 80% acetonitrile containing 0.1% formic acid over 120 min at a flow rate of 300 nl min^−1^. The MS survey scans were acquired at a resolution of 60 000 over a mass range of *m*/*z* 350–1800. In each cycle, data‐dependent MS2 scans were acquired for 3 sec. The isolation of precursors was performed in the quadrupole with a window of 2.0 *m*/*z*. The singly charged ions and ions with four or more charges were excluded for MS/MS analysis. The dynamic exclusion time was set to 40 sec. The MS/MS scans were performed in the ion trap after collision induced dissociation (35%). The target value for the full scan MS scan was 4 × 10^5^ with a maximum injection time of 50 ms, and that for the MS/MS scan was 1 × 10^4^ with a maximum injection time of 50 msec. All LC‐MS/MS data were processed using Proteome Discoverer (version 2.5; Thermo Fisher Scientific). Searches were done against the *Zea mays* sequences in the NCBI database (version March 2021, 156 326 sequences) concatenated with a database of common contaminants often found in proteomics experiments. The detailed bioinformatic processing of identified proteins is given in the Supplementary Information.

### Soil microbial community analysis

DNA was extracted from rhizosphere soil samples (2 g each) collected from the field and greenhouse experiment, using the RNeasy® PowerSoil Total RNA Kit and a separate DNA Elution Kit (Qiagen Inc. MD, USA) according to the manufacturer's protocol. The quality and quantity of the obtained DNA were measured with the Qubit dsDNA HS assay (Life Technologies, Austin, TX, USA). The DNA extract was diluted to 1 ng μl^−1^ before being used as a template for polymerase chain reaction (PCR) amplification. Illumina MiSeq amplicon sequencing, targeting 16S rRNA and ITS2, was used to characterize the bacterial and fungal microbial communities, respectively (Kozich et al., 2013) (see Supplementary Information for primer sequence and PCR conditions). All PCR products were checked on 1% agarose gel to ensure the amplification was successful. Finally, PCR products of bacteria and fungi were pooled to create a DNA library before sequencing. The concentration of the library was quantified with both Qubit dsDNA HS assay and quantitative PCR as described in Kozich et al. (2013). The insert sizes of the library were determined with the Agilent 2100 Bioanalyzer (Agilent Technologies, Santa Clara, CA, USA). Illumina MiSeq sequencing was performed on an Illumina MiSeq 4000 (Illumina, San Diego, CA, USA) at Clemson University (Lim et al., 2019). The detailed bioinformatic processing of sequence data is given in the Supplementary Information.

### Statistical analysis

Three‐way anova was used to determine the effects of drought, soil type, rhizobiome inoculation, and their interactions on all plant morphological, physiological, and biochemical properties. Student's *t*‐test was used to assess the significant differences of peak intensity of proteins between different treatments. A protein was considered statistically significant only when *P* < 0.05, and a fold‐change of ≥2 or ≤−2 in at least one or two biological replicates. The profiles and variances of foliar primary metabolites and root proteomics were visualized in heatmaps with hierarchical clustering and PCA at MetaboAnalyst 4.0 platform (Chong et al., 2018). To measure the effect size and significances on the beta‐diversity in soil microbial communities, Principal coordinate analysis and the significance test (PERMANOVA test, Adonis tool) with 999 permutations based on Bray–Curtis distance were performed in the “vegan” package on the R platform (Strati et al., 2017). Hierarchical cluster analysis at the family level across all samples was performed in R using commands in the “pheatmap” package and plotted with the heatmap (Li et al., 2017). The LEfSe method was performed to identify bacterial and fungal taxa with significantly different abundances between groups (Segata et al., 2011). The Kruskal–Wallis sum‐rank test (*P* < 0.05) was used in the LEfSe analysis to detect features with significantly different abundances between the specified categories, and this was followed by a linear discriminant analysis to estimate the effect size of each differentially abundant feature (logarithmic linear discriminant analysis score >4.0) through the on‐line Galaxy platform (http://huttenhower.sph.harvard.edu/galaxy).

## AUTHOR CONTRIBUTIONS

NT in consultation with ZLZ and VS concieved the study, ZLZ, BJ, VS, and NT planned and designed the experiments. ZLZ conducted the main greenhouse experiment, BJ, BC, JG and NT performed experiments and data analysis. ZLZ wrote the first draft of the manuscript and all authors contributed to revisions.

### OPEN RESEARCH BADGES

This article has earned an Open Data badge for making publicly available the digitally‐shareable data necessary to reproduce the reported results. The 16S rRNA gene and fungal ITS amplicon sequences are available in the NCBI database underthe BioProject accession number PRJNA839620, Biosample IDs SAMN28544845‐SAMN28544942, and experiment IDs SRX15369961‐SRX15370058. The metabolomics data set has been deposited to MassIVE Repository (MassIVEMSV000089513). Proteomics dataset has been deposited to MassIVE Repository (MassIVE MSV000089461).

## Supporting information


**Notes S1.** Additional materials and methods: (1) primer sequence information and PCR conditions; (2) bioinformatic processing of sequence data; (3) bioinformatic processing of identified proteins from root proteomics.
**Figure S1.** Schematic diagram of the experimental design.
**Figure S2.** Water stress status for drought and ambient treatments.
**Figure S3.** Peak areas of proline and soluble sugars in maize leaves.
**Figure S4.** Abundance and pattern of 23 phytohormones in roots of maize.
**Figure S5.** Principal coordinate analysis of bacterial and fungal communities in the rhizosphere soil of maize across different treatments.
**Table S1.** Nutrient information of soil inocula before inoculation.
**Table S2.** Results of three‐way anova for variables evaluating drought effect.

## Data Availability

The authors declare that the data supporting the findings of this study are available within the article and its Supplementary Information file, and from the corresponding author on request. The 16S rRNA gene and fungal ITS amplicon sequences are available in the NCBI database under the BioProject accession number PRJNA839620, Biosample IDs SAMN28544845‐SAMN28544942, and experiment IDs SRX15369961‐SRX15370058. The metabolomics data set has been deposited to MassIVE Repository (MassIVE MSV000089513). Proteomics dataset has been deposited to MassIVE Repository (MassIVE MSV000089461).
