# Supplementary material for: Cross‐inoculation of rhizobiome from a congeneric ruderal plant imparts drought tolerance in maize (Zea mays) through changes in root morphology and proteome
Source: Plant J. 2022 Jun 8;111(1):54–71. doi: 10.1111/tpj.15775 (PMC9542220; doi:10.1111/tpj.15775)
Supplement: Supplementary file 1 — Notes S1. Additional materials and methods: (1) primer sequence information and PCR conditions; (2) bioinformatic processing of sequence data; (3) bioinformatic processing of identified proteins from root proteomics. Figure S1. Schematic diagram of the experimental design. Figure S2. Water stress status for drought and ambient treatments. Figure S3. Peak areas of proline and soluble sugars in maize leaves. Figure S4. Abundance and pattern of 23 phytohormones in roots of maize. Figure S5. Principal coordinate analysis of bacterial and fungal communities in the rhizosphere soil of maize across different treatments. Table S1. Nutrient information of soil inocula before inoculation. Table S2. Results of three‐way anova for variables evaluating drought effect. [file TPJ-111-54-s001.docx]

**Supporting Information for:**

**Cross inoculation of rhizobiome from a congeneric ruderal plant imparts drought tolerance in maize (*Zea mays*)**

Ziliang Zhang^1^, Bhupinder Singh Jatana^1^, Barbara Campbell^2^, Jasmine Gill^1^, , Vidya Suseela^1*^ Nishanth Tharayil^1*^,

^1^ Department of Plant & Environmental Sciences, Clemson University, Clemson, South Carolina

^2^ Department of Biological Sciences, Clemson University, Clemson, South Carolina

The following Supporting Information is available for this article:

**Notes S1. Additional materials and methods** (1) Primer sequence information and PCR conditions; (2) Bioinformatic processing of sequence data; (3) Bioinformatic processing of identified proteins from root proteomics

**Fig. S1.** Schematic diagram of the experimental design.

**Fig. S2.** Water stress status for drought and ambient treatments.

**Fig. S3.** The peak areas of proline and soluble sugars in maize leaves.

**Fig. S4.** Abundance and pattern of 23 phytohormones in roots of maize

**Fig. S5.** Principal coordinate analysis of bacterial and fungal communities in the rhizosphere soil of maize across different treatments.

**Table S1.** Nutrient information of soil inocula before inoculation.

**Table S2.** Results of three-way ANOVA for variables evaluating drought effect.

**Notes S1 Additional materials and methods**

***Primer sequence information and PCR conditions***

Bacterial primers for and amplification of the V4 region of the 16S rRNA gene as well as library preparation were described previously (Deaver *et al.,* 2020). Briefly, dual barcoded primers were used spanning the V4 region as described (Kozich *et al.,* 2013) in a 30-cycle PCR with Phusion high fidelity DNA polymerase (ThermoFisher), but with a slightly modified R primer (Apprill et al, 2015). For the fungal ITS region, primers routinely used for ITS amplification (ITS1f, ITS2) (Gardes and Bruns, 1993) were modified with dual barcodes as described in Kozich et al. (2013) and O’Connell et al. (2020). PCR conditions for the ITS region were the same as above, but with an annealing temperature of 57 °C instead of 61 °C. The sizes of the ITS region were between 263-431 bp after trimming of non-ITS sequences.

***Bioinformatic processing of sequence data***

Preliminary screening of the raw sequence data resulted in trimming approximately 15 bp from the 5’ and 20-50 bp from 3’ end of the sequence to remove low-quality bases prior to joining. The sequences were then analyzed using the latest Qiime2 workflow (Bolyen *et al.,* 2019). Briefly, amplicon sequence variants (ASVs) were determined in Qiime2 via the DADA2 pipeline using an open reference picking mode, based on no more than 1% difference between sequences; and likely represents strains to species level variation (Callahan *et al.,* 2016). Sequences that occurred less than ten times in the entire dataset were removed using the filter table option. Taxonomic classification of the bacterial non-chimeric, denoised, joined reads was performed using a Qiime2 classifier (sk-learn, Pedregosa *et al.,* 2011) pre-trained on Silva v123 at 99% identity (Bokulich *et al.,* 2018). Representative bacterial ASV sequences were further analyzed by multiple sequence alignment via MAFFT and FastTree to reconstruct the phylogenetic tree prior to beta diversity analysis (Katoh *et al.,* 2002). Fungal ITS sequence variants (expected ITS sizes) were determined similarly (joining, DADA2 pipeline), but with no phylogenetic analyses and the use of the UNITE version 7.2 (UNITE‐Community, 2017) as the taxonomic classifier. Bacterial and fungal sequences per soil sample were rarefied to 13,000 and 19,000 sequences, respectively, prior to beta diversity analyses. ASV tables at different taxonomic levels for data analyses were exported from Qiime2 and used in statistical analyses.

***Bioinformatic processing of identified proteins from root proteomics.***

All LC−MS/MS data were processed using Proteome Discoverer (Thermo Fischer Scientific, version 2.5). A pair of missed tryptic cleavages were allowed, and the minimum peptide length was set to six amino acids. Variable modifications included oxidation (M) and protein N-terminal acetylation and deamidation (N, Q). Fixed modification included carbamidomethylation (C). MS and MS/MS ion tolerances were set at 10 ppm and 0.5 Da, respectively. The false-discovery rate (FDR) was estimated using the integrated Percolator tool. Only protein hits identified with a 1% FDR threshold, at least 10 percent of protein sequence covered by identified peptide, one peptide sequence unique to the protein group, and medium and high peptide confidence were accepted. For protein quantification, the Sequest HT was used. We accepted most of the default settings in the Proteome Discoverer software 2.5 In addition, the 1% FDR was required at the peptide and protein levels. Label-free quantification was performed using Minora Feature Detector followed by Feature mapper and Precursor Ions Quantifier node. Differentially abundant proteins (DAPs) were filtered based on the log2 fold change

threshold set to 2 (DAPs with log2 fold change ≥ 2 or ≤−2 were considered as up- and down-regulated, respectively). The DAPs were categorized according to their Gene Ontology (GO) annotations for Biological Process and Molecular Function categories (http://www.geneontology.org) to find significant enrichment GO entries by comparing significantly different proteins with the overall identified proteins as background (Zeng *et al.,* 2019; Ebinezer *et al.,* 2020). The metabolic pathway analysis of the proteins was conducted according to the Kyoto Encyclopedia of Genes and Genomes (KEGG) Pathway Database (https://www.genome.jp/kegg/pathway.html). GO and KEGG pathway enrichment were performed with Benjamini−Hochberg false discovery rate (FDR) significance threshold set at 0.05. GO enrichment and KEGG pathway mapping were performed using ‘clusterProfiler’ package in R (Yu et al., 2012).


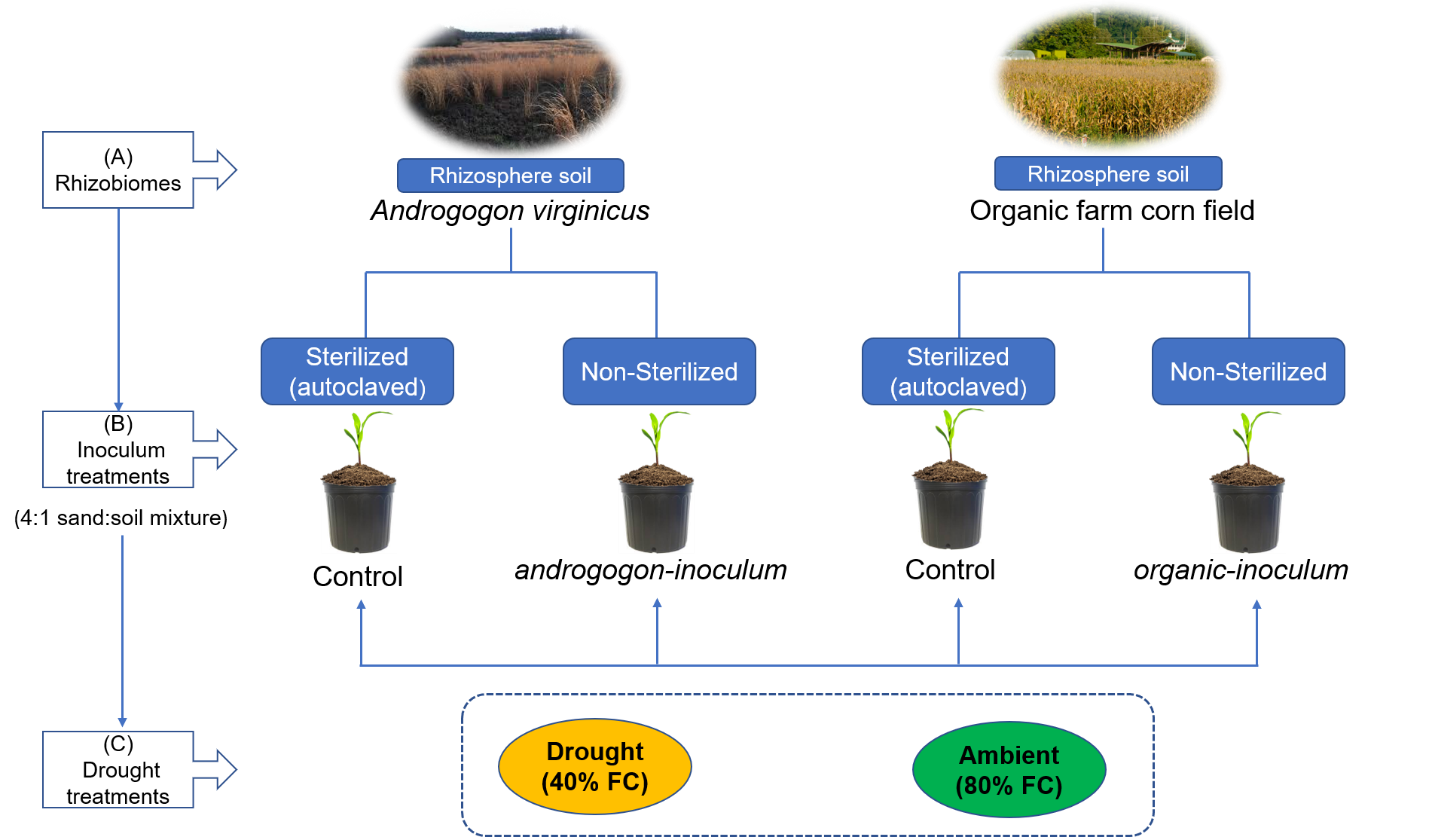


**Fig. S1**. Schematic diagram of the experimental design including rhizobiome collection (A), inoculum treatments (B), and drought treatments (C). FC: field capacity.

**Fig. S2.** Water stress status (Ambient (80% field capacity) and drought stress (40% field capacity) ) of maize monitored using time-domain reflectometry (TDR) probes. Drought induction was started 20 days after sowing the seeds. Before drought application, the soil moisture of all pots was maintained at 60% field capacity. A, non-sterilized *andropogon-inoculum*; O, non-sterilized *organic-inoculum*; A-A, Sterilized *andropogon-inoculum*; A-O, sterilized *organic-inoculum*.

**Fig. S3.** The peak areas of proline (a) and soluble sugars (b-d) in maize leaves detected using targeted primary metabolites profiling in GC-MS. Error bars are ±1SE of the mean (n = 5) with the asterisk indicating significant differences between drought and ambient treatments at *P* < 0.05.


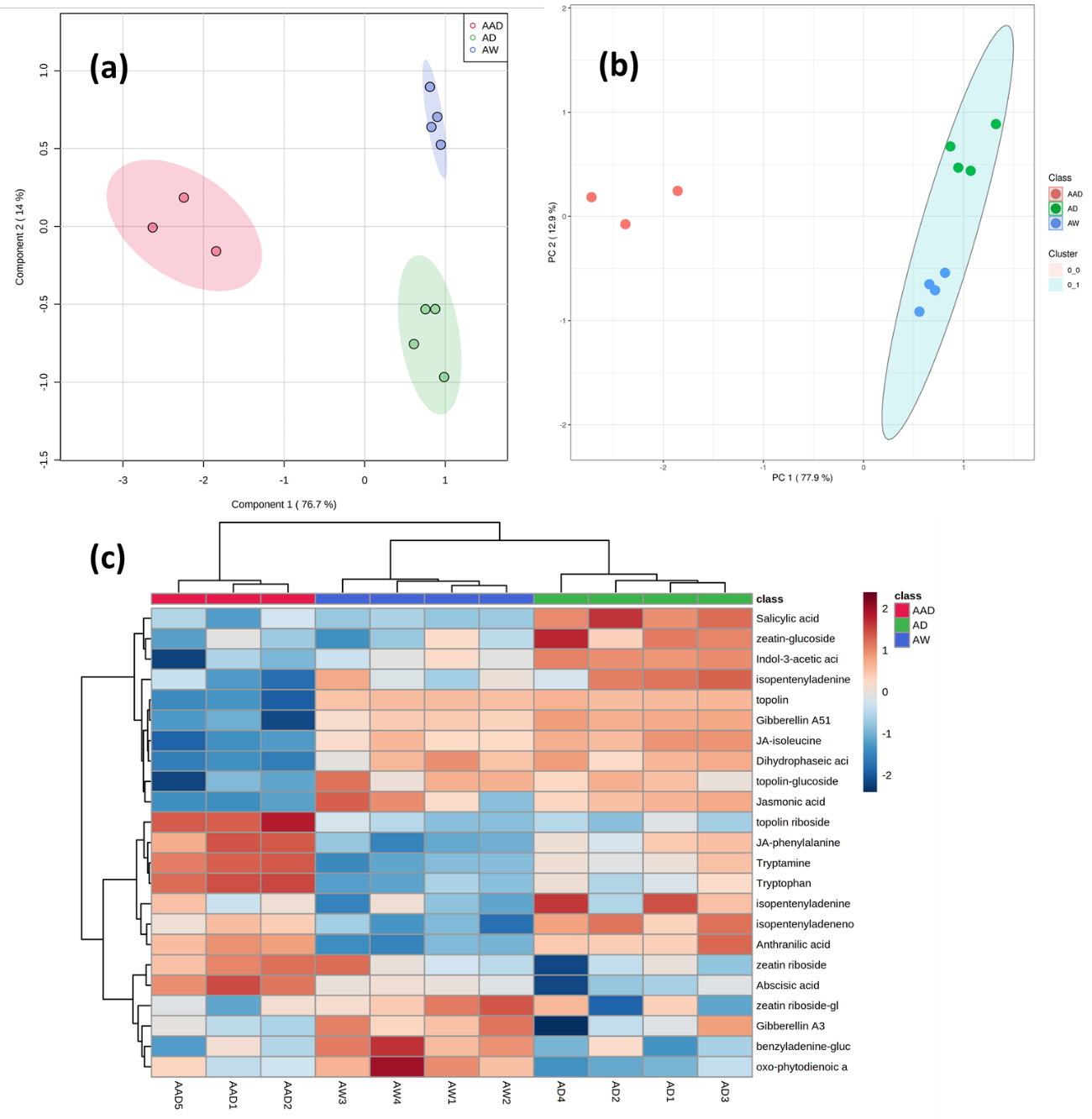


**Fig. S4.** Principal component analysis (a), self-organizing map (b) and heatmap with hierarchical clustering (c) of 23 phytohormones detected in corn root subjected to various treatments. ; AAD: sterilized *andropogon-inoculum* under drought condition; AD: non-sterilized *andropogon-inoculum* under drought condition; AW: non-sterilized *andropogon-inoculum* under ambient condition.

**
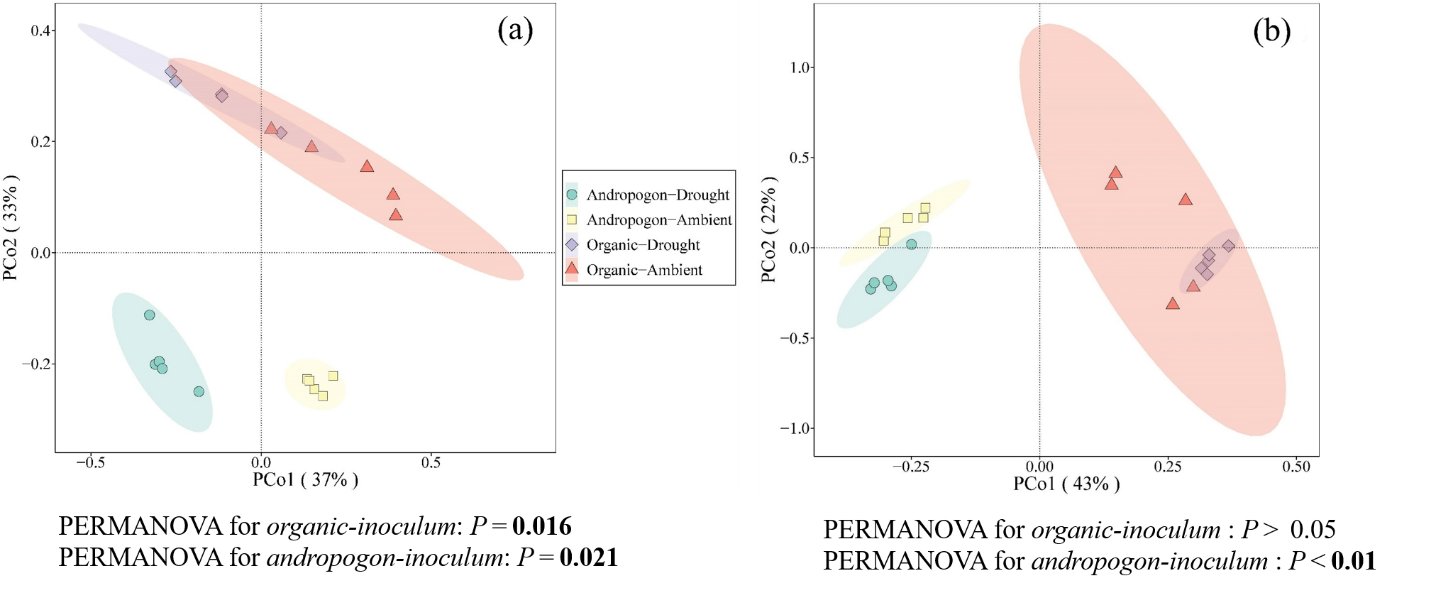
Fig. S5.** Principal coordinate analysis (PCoA) plot based on Bray-Curtis distances of bacterial (a) and fungal (b) communities in the rhizosphere soil of maize across different treatments. Andropogon-Drought: non-sterilized *andropogon-inoculum* under drought condition; Andropogon-Ambient: non-sterilized *andropogon-inoculum* under ambient condition; Organic-Drought: non-sterilized *organic-inoculum* under drought condition; Organic-Ambient: non-sterilized *organic-inoculum* under ambient condition.

**Table S1.** Concentrations of available nitrogen (N), phosphorus (P), and potassium (K) in *andropogon-inoculum* and *organic-inoculum* before inoculation

|  | N (mg kg^-1^) | P (mg kg^-1^) | K (mg kg^-1^) |
| --- | --- | --- | --- |
| *andropogon-inoculum* | 525 | 153.5 | 233.5 |
| *organic-inoculum* | 275 | 312.5 | 248.6 |

**Table S2.** Results of three-way ANOVA showing the *P* values for responses of shoot biomass, root biomass, net photosynthetic rate (Pn), stomatal conductance (Gc), Malondialdehyde (MDA) content, superoxide dismutase (SOD), peroxidase (POD), catalase (CAT), specific root length (SRL) and specific root surface area (SRSA) to drought (D), soil type (S), and rhizobiome inoculation (Ri). *P* values less than 0.05 are in bold

| Factors | Drought | Soil type | Rhizobiome inoculation | D × S | D × Ri | S × Ri | D × S × Ri |
| --- | --- | --- | --- | --- | --- | --- | --- |
| Shoot biomass | <**0.001** | **<0.001** | 0.782 | 0.633 | **0.007** | **<0.001** | 0.770 |
| Root biomass | <**0.001** | **0.001** | 0.819 | 0.728 | **0.050** | **0.003** | 0.519 |
| Pn | <**0.001** | **0.002** | <**0.001** | 0.173 | 0.310 | 0.742 | 0.800 |
| Gs | **0.001** | **0.036** | **0.003** | 0.139 | 0.224 | 0.228 | 0.964 |
| MDA | <**0.001** | **0.047** | **0.012** | **<0.001** | 0.365 | **0.008** | **0.044** |
| SOD | <**0.001** | **0.014** | **0.005** | **0.032** | 0.281 | 0.055 | 0.097 |
| CAT | **0.001** | **0.050** | **0.041** | **0.003** | **0.045** | 0.089 | **0.023** |
| POD | **0.020** | **0.028** | **<0.001** | **0.012** | **0.055** | 0.221 | 0.352 |
| SRL | <**0.001** | 0.209 | <**0.001** | **0.019** | <**0.001** | **0.006** | <**0.001** |
| SRSA | <**0.001** | 0.212 | <**0.001** | 0.112 | <**0.001** | **0.038** | **0.027** |

**References**

**Apprill A, McNally S, Parsons R, Weber L.** 2015. Minor revision to V4 region SSU rRNA 806R gene primer greatly increases detection of SAR11 bacterioplankton. Aquatic Microbial Ecology **75(2)**, 129-137.

**Bolyen E, Rideout JR, Dillon MR, Bokulich NA, Abnet CC, Al-Ghalith GA, Alexander H, Alm EJ, Arumugam M, Asnicar F.** 2019. Reproducible, interactive, scalable and extensible microbiome data science using QIIME 2. Nature Biotechnology **37**, 852-857.

**Bokulich NA, Kaehler BD, Rideout JR, Dillon M, Bolyen E, Knight R, Huttley GA, Caporaso JG.** 2018. Optimizing taxonomic classification of marker-gene amplicon sequences with QIIME 2’s q2-feature-classifier plugin. Microbiome **6(1)**, 90.

**Callahan BJ, McMurdie PJ, Rosen MJ, Han AJ, Johnson AJA, Holmes SP.** 2016. DADA2: high-resolution sample inference from Illumina amplicon data. Nature Methods **13(7)**, 581-583.

**Deaver JA, Diviesti KI, Soni M, Campbell BJ, Finneran KT, Popat SC**. 2020. Palmitic acid accumulation limits methane production in anaerobic co-digestion of fats, oils and grease with municipal wastewater sludge. Chemical Engineering Journal **125235**.

**Ebinezer LB, Franchin C, Trentin AR, Carletti P, Trevisan S, Agrawal GK, Rakwal R, Quaggiotti S, Arrigoni G, Masi A.** 2020. Quantitative Proteomics of Maize Roots Treated with a Protein Hydrolysate: A Comparative Study with Transcriptomics Highlights the Molecular Mechanisms Responsive to Biostimulants. Journal of Agricultural and Food Chemistry **68**, 7541-7553.

**Gardes M, Bruns TD.** 1993. ITS primers with enhanced specificity for basidiomycetes‐application to the identification of mycorrhizae and rusts. Molecular Ecology **2(2)**, 113-118.

**Katoh K, Misawa K, Kuma KI, Miyata T.** 2002. MAFFT: a novel method for rapid multiple sequence alignment based on fast Fourier transform*.* Nucleic Acids Research **30(14)**, 3059-3066.

**Kozich JJ, Westcott SL, Baxter NT, Highlander SK, Schloss PD.** 2013. Development of a dual-index sequencing strategy and curation pipeline for analyzing amplicon sequence data on the MiSeq Illumina sequencing platform. Applied and Environmental Microbiology **79(17)**, 5112-5120.

**O’Connell LM, Santos R, Springer G, Burne RA, Nascimento MM, Richards VP.** 2020. Site-specific profiling of the dental mycobiome reveals strong taxonomic shifts during progression of early-childhood caries. Applied and Environmental Microbiology **86(7)**.

**Pedregosa F, Varoquaux G, Gramfort A, Michel V, Thirion B, Grisel O, Blondel M, Prettenhofer P, Weiss R, Dubourg V, Vanderplas J.** 2011**.** Scikit-learn: Machine learning in Python. The Journal of Machine Learning Research **12**, 2825-2830.

**UNITE‐Community.** 2017**.** UNITE QIIME release. Version 01.12. 2017.

**Yu GG, Wang LG, Han YY, He QY.** 2012. clusterProfiler: an R package for comparing biological themes among gene clusters. Omics: a journal of integrative biology **16(5)**, 284-287.

**Zeng W, Peng YL, Zhao XQ, Wu BY, Chen FQ, Ren B, Zhuang ZL, Gao QH, Ding YF.** 2019. Comparative proteomics analysis of the seedling root response of drought-sensitive and drought-tolerant maize varieties to drought stress. International Journal of Molecular Sciences **20(11)**, 2793.
